# Clinical and molecular landscape of paediatric cerebral and spinal cavernous malformations

**DOI:** 10.1093/braincomms/fcaf408

**Published:** 2025-10-30

**Authors:** Sandro Benichi, Estelle Balducci, Joseph Benzakoun, Boulos Ghannam, Christian Attieh, Alice Metais, Marie Bourgeois, Lelio Guida, Sophie Kaltenbach, Arnault Tauziede-Espariat, Florence Riant, Patrick Villarese, Coline Lefevre, Charles-Joris Roux, Stéphanie Puget, Olivier Naggara, Catherine Oppenheim, Rima Nabbout, Nathalie Boddaert, Kévin Beccaria, Elisabeth Tournier-Lasserve, Manoëlle Kossorotoff, Pascale Varlet, Vahid Asnafi, Guillaume Canaud, Thomas Blauwblomme

**Affiliations:** Department of Pediatric Neurosurgery, Necker – Enfants Malades Hospital, Paris 75015, France; Paris-Cité University, Translational Medicine and Targeted Therapies Laboratory, UMR 1151, Necker – Enfants Malades Institute, Paris 75015, France; National Center for Pediatric Stroke CNRAVC, Paris 75015, France; French Reference Center for Rare Brain and Eye Vascular Malformations CERVCO, Paris 75015, France; Department of Onco-Hematology, Necker – Enfants Malades Hospital, Paris 75015, France; Department of Neuroradiology, GHU-Paris Psychiatrie et Neurosciences, Hôpital Sainte Anne, Paris 75014, France; Institute of Psychiatry and Neuroscience of Paris (IPNP), Université Paris Cité, Paris 75014, France; Department of Pediatric Neurosurgery, Necker – Enfants Malades Hospital, Paris 75015, France; Department of Pediatric Neurosurgery, Necker – Enfants Malades Hospital, Paris 75015, France; Institute of Psychiatry and Neuroscience of Paris (IPNP), Université Paris Cité, Paris 75014, France; Department of Neuropathology, GHU-Paris Psychiatrie et Neurosciences, Paris F-75014, France; Department of Pediatric Neurosurgery, Necker – Enfants Malades Hospital, Paris 75015, France; Department of Pediatric Neurosurgery, Necker – Enfants Malades Hospital, Paris 75015, France; Department of Onco-Hematology, Necker – Enfants Malades Hospital, Paris 75015, France; Institute of Psychiatry and Neuroscience of Paris (IPNP), Université Paris Cité, Paris 75014, France; Department of Neuropathology, GHU-Paris Psychiatrie et Neurosciences, Paris F-75014, France; Université Paris Cité, AP-HP Department of Medical Genetics, Saint Louis Hospital, Paris 75010, France; Department of Onco-Hematology, Necker – Enfants Malades Hospital, Paris 75015, France; Department of Onco-Hematology, Necker – Enfants Malades Hospital, Paris 75015, France; Department of Pediatric Radiology, Necker – Enfants Malades Hospital, Paris 75015, France; Department of Neurosurgery, Fort-de-France 97261, France; National Center for Pediatric Stroke CNRAVC, Paris 75015, France; French Reference Center for Rare Brain and Eye Vascular Malformations CERVCO, Paris 75015, France; Department of Neuroradiology, GHU-Paris Psychiatrie et Neurosciences, Hôpital Sainte Anne, Paris 75014, France; Institute of Psychiatry and Neuroscience of Paris (IPNP), Université Paris Cité, Paris 75014, France; Department of Neuroradiology, GHU-Paris Psychiatrie et Neurosciences, Hôpital Sainte Anne, Paris 75014, France; Institute of Psychiatry and Neuroscience of Paris (IPNP), Université Paris Cité, Paris 75014, France; Department of Pediatric Neurology, Necker – Enfants Malades Hospital, Paris 75015, France; Department of Pediatric Radiology, Necker – Enfants Malades Hospital, Paris 75015, France; Department of Pediatric Neurosurgery, Necker – Enfants Malades Hospital, Paris 75015, France; Université Paris Cité, AP-HP Department of Medical Genetics, Saint Louis Hospital, Paris 75010, France; National Center for Pediatric Stroke CNRAVC, Paris 75015, France; French Reference Center for Rare Brain and Eye Vascular Malformations CERVCO, Paris 75015, France; Department of Pediatric Neurology, Necker – Enfants Malades Hospital, Paris 75015, France; Institute of Psychiatry and Neuroscience of Paris (IPNP), Université Paris Cité, Paris 75014, France; Department of Neuropathology, GHU-Paris Psychiatrie et Neurosciences, Paris F-75014, France; Department of Onco-Hematology, Necker – Enfants Malades Hospital, Paris 75015, France; Paris-Cité University, Translational Medicine and Targeted Therapies Laboratory, UMR 1151, Necker – Enfants Malades Institute, Paris 75015, France; Department of Pediatric Neurosurgery, Necker – Enfants Malades Hospital, Paris 75015, France; National Center for Pediatric Stroke CNRAVC, Paris 75015, France; French Reference Center for Rare Brain and Eye Vascular Malformations CERVCO, Paris 75015, France

**Keywords:** vascular malformation, paediatric intracranial haemorrhage, stroke, brain mosaicism, brain probability atlas

## Abstract

We aimed to decipher the natural history of paediatric cerebral and intraspinal medullary cavernous malformations and their genetic background, with an emphasis on the effect of mutational burden on their radiological features and haemorrhagic risk. We retrospectively collected clinical and radiological data and the natural history of the disease during the follow-up of all consecutive patients with cavernous malformations over the last 2 decades at a unique paediatric centre. We created an MRI atlas after lesion segmentation and normalization to perform voxel-based lesion symptom mapping. Targeted germline DNA sequencing of CCM1-3 genes and somatic lesions and targeted sequencing of RAS and PI3 K pathway genes were performed. In total, 257 patients carrying 786 brain and 14 spinal cavernous malformations were analysed. Age at diagnosis was 9.5 ± 5 years. Follow-up data were available for 718 lesions with a mean follow-up of 5.59 ± 3.28 years. Then, 134 brain (80 with focal onset seizures) and 10 spinal cavernous malformations were surgically excised. Furthermore, 95 of the/144 DNA extracted from lesions were successfully sequenced for somatic variants (*PIK3CA*, *MAP3K3*, *KRIT1* and *KRAS*). We found an annual haemorrhagic risk of 1.9%, which was increased by type (sporadic or postradiation), location (brainstem or spinal), and radiological parameters (extracapsular haemorrhage, volume, signal, or presence of developmental venous anomaly). Cavernous malformations symptoms and radiological features were highly dependent on brain location. Surgical risk for cortical brain lesions was 0.9%, with 85% international league against epilepsy score grade of 1–2. Furthermore, 62% of operated lesions carried a pathogenic *PIK3CA* variant. The *MAP3K3* somatic variant alone or in association with a *PIK3CA* variant decreased the annual haemorrhagic risk by 4.8 ± 1.5 (*P* < 0.05). We confirmed that 96% of cavernous malformations that are satellites of a developmental venous anomaly carried a *PIK3CA* variant and increased the relative haemorrhagic risk by 3 ± 0.31 (*P* = 3.8.10^−3^). We report an annual haemorrhagic risk of 1.9%, which is modulated by radiological features (i.e. extracapsular haemorrhage, volume, signal, and location) and lesion type. Here, we describe the first MRI atlas. Surgery yields excellent general outcomes in supratentorial superficial lesions, unlike deep-seated cavernous malformations, which is associated with high morbidity. Molecular diagnosis of operated lesions highlighted *PIK3CA* as a factor associated with aggressiveness in both sporadic and familial cavernous malformations. *MAP3K3* decreased the risk. Finally, we confirmed that developmental venous anomaly is a radiological landmark of *PIK3CA*.

## Teaser

*Benichi et al.* studied the natural history of paediatric brain cavernous malformations. Using MRI and genetic sequencing of the lesions, the authors identified imaging markers (volume, signal, location) and genetic factors (*PIK3CA* gene variant) associated with an increased risk of haemorrhage.

## Graphical Abstract

**Graphical Abstract:**
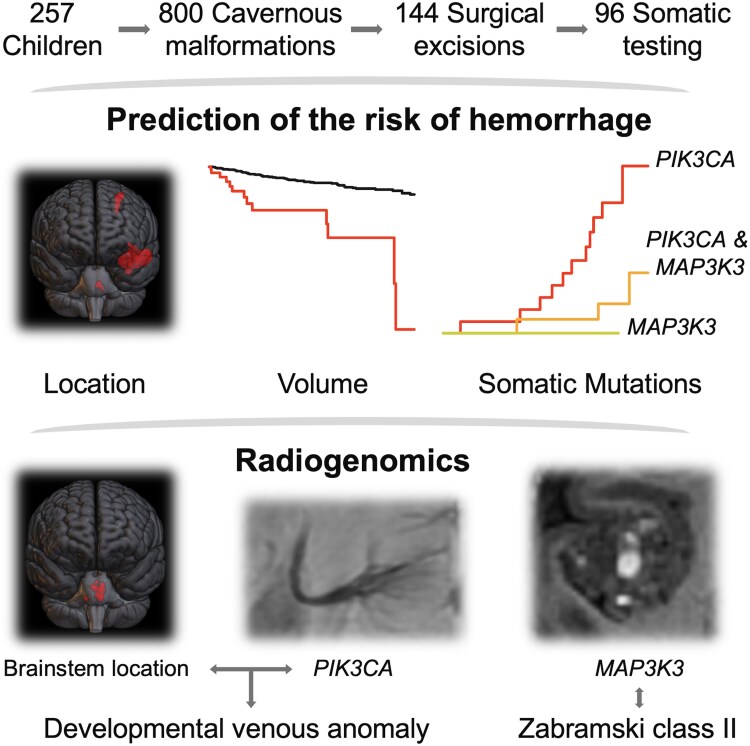

## Introduction

### State-of-the-art

Cavernous malformation (CM) of the central nervous system is a vascular malformation histologically defined as a pseudotumoural proliferation of dilated capillaries, in ‘cavern’ shape, without interposition of brain tissue.^1,2^ The prevalence of CM is approximately 1 in 200 individuals in the general population, and its incidental finding is lower in the paediatric population (0.2%).^3,4^ They can be sporadic (S-CM) or familial (F-CM) through highly penetrant predisposing germline mutation of one of the three cerebral cavernous malformation (CCM) genes: Krev interaction trapped protein 1 (*KRIT1* = *CCM1)*, Malcavernin (*MGC4607* = CCM2), and programmed cell death 10 (*PDCD10* = *CCM3*), a condition called cavernomatosis.^5-8^ Therefore, CM can occur after radiation beam therapy (RBT-CM) for brain tumour.^7^ CM pathophysiology was described during the last 2 decades, primarily because of the study of familial forms. The main mechanism relies on the upregulation of the MAP/ERK kinase 3 (MEKK3) pathway.^9-12^ CM arises from the dysregulation in the crosstalk mechanisms of remodelling, particularly intussusceptive angiogenesis, between endothelial cells, pericytes, and surrounding astrocytes.^13-16^ CM grows through the expansion of clonal endothelial cells, undergoing an endothelial-to-mesenchymal fate transition and recruiting surrounding wild-type cells in a pseudotumoural way.^10,12^ Dysfunction of the brain endothelial cell–cell interaction by disruption of the cell-adherent junction complexes coupled with pro-angiogenic factors from pericytes and astrocytes will lead to blood–brain barrier dysfunction, leakage, inflammation, and gliosis.^3,16^ This latter phenomenon triggers intra- or extra-lesional haemorrhage and subsequent clinical symptoms: seizure, neurological deficit, and intracranial hypertension.

CM accounts for 17% of paediatric intracranial haemorrhage.^17,18^ The annual haemorrhagic risk varies from 1% to 25% in different studies, depending on the radiological features evaluated by the Zabramski score, mainly studied in adult series.^1,19-23^ Surgical treatment is required in cases where CM-related symptoms include focal-onset seizure, intracranial haemorrhage, and neurological deficit.^24^ Growing evidence shows that the course of CM can range from quiescent to highly aggressive lesions.^25^ Outcomes depend on CM location^26^; deep-seated and brainstem CM carry significant morbidity on their own, along with increased surgical risks.^27-29^ More recently, molecular biology insights suggested that somatic gain-of-function mutations of the phosphatidylinositol 3-kinase, catalytic, alpha (*PIK3CA*) in CM increase its aggressiveness.^12,30^

Previous studies have focused on the clinical course or molecular landscape of this disease; however, they have rarely provided extensive translational data. Thus, data on genotype–phenotype correlation remain limited.^31-33^ In the paediatric population, only a few patients are available, with a preponderance of familial form; overview of the molecular landscape is still lacking.^21,26,27,34-38^

### Objectives of the study

This study adopted a clinical, radiological, and molecular approach to decipher the natural history of paediatric cerebral and intraspinal medullary (ISM) CM. We report their radiological features and risk factors and treatment modalities and outcomes, with a focus on epilepsy management. Furthermore, their molecular features and correlation between mutational burden and haemorrhagic risk were investigated.

## Materials and methods

### Study design, setting, and participants

This single-centre retrospective study included all children aged <18 years diagnosed with intra-axial CM between January 01, 2004, and May 31, 2023.

We followed the Strengthening the Reporting of Observational Studies in Epidemiology guidelines for a retrospective study.^39^ In this noninterventional study of routinely acquired data, individual written consent was waived. Patients (or parents depending on patient age) who underwent CCM gene screening and/or tissue sample somatic panel sequencing provided written consent and/or nonopposition consent according to local regulations for biomedical research. The biocollection is registered under number DC-2020-3940 by the Ministry of Research. A commitment to compliance was submitted to the French National Agency for Informatics and Liberties (number 2233665v0). This study was conducted according to the Helsinki Declaration for Human Rights.

Exhaustive query of all consecutive patients was performed using our local data warehouse, Dr. Warehouse.^40^ We included children with a radiological diagnosis of CM when it was visible on gradient echo and T1-weighted imaging. We defined S-CM as a unique CM without a familial history or multiple CM satellites of developmental venous anomalies (DVAs). We defined F-CM as one or more CM with a family history of CM or *de novo* multiple unrelated CM. We defined post-radiation beam therapy (RBT) CM (RBT-CM) as CM that appeared on follow-up imaging after RBT but was absent on initial imaging. We excluded patients for whom initial MRI scans were unavailable. Fig. S1 presents the flowchart. Clinical parameters at diagnosis and follow-up were as follows: date of birth, age at diagnosis, initial symptom, type of seizure, electroencephalogram pattern, treatment, comorbidity, surgical treatment, age at surgery, academic results, neurological assessment, epilepsy control and treatment, and last follow-up. The radiological initial parameters were as follows: volume, normalized signal intensity and standard deviation, Zabramski score,^19,21^ number of CM, location of CM, and associated DVA. Haemorrhage was defined as homogenous hyperdensity on CT (when available) or hypersignal T1 and hypersignal on gradient echo sequences on MRI with mass effect and/or perilesional oedema. Radiological data were collected during follow-up (i.e. new CM, new haemorrhage assessed by volumetric growth of CM, and Zabramski score modification). The interval between MRI scans varied depending on the neurosurgeons in charge, ranging from 1 to 5 years and in cases of intercurrent symptoms requiring imaging. Radiological data of CM were recorded on the first MRI scan, and CM haemorrhage was assessed by comparing all available MRI scans. Haemorrhage was defined through qualitative assessment and confirmed by volume measurement.

### MRI postprocessing and voxel-based lesion symptom mapping analysis

The manual CM segmentation was performed by a single operator (SB) using MRICroGL. All 3D imaging (3D T1 MRI with or without contrast enhancement) procedures were then rigidly co-registered to the standard MNI-152 1-mm T1 template using the FSL toolbox (‘https://fsl.fmrib.ox.ac.uk/fsl/fslwiki’), as previously described.^41^ We performed VLSM, a method that associates behaviour with lesion sites on a voxel-by-voxel basis on binary parameters, using the NiiStat toolbox (‘https://github.com/neurolabusc/NiiStat.git’) for Matlab©. VLSM involves overlaying individual lesion maps onto a common template and applying a univariate logistic regression model at each voxel to determine whether the presence or absence of a lesion in that voxel is associated with a specific parameter across a group of individuals.^42,43^  Fig. S2 presents the main steps of brain and lesion volume normalization and VLSM.

The lesion analysis was performed using Liebermeister quasi-exact test with 1000 permutations, including voxels shared by at least two individuals, and the significance level was set at *P* < 0.05. To test the unsupervised radiological parameters of CM, CM volume, CM signal homogeneity (assessed by signal standard deviation), and CM signal intensity (assessed by the mean signal) were computed after normalization of MR signal and z scoring correction for the injection status. CM extracapsular haemorrhage (ECH) was assessed by a single operator (SB) as previously described.^21^

### Germline DNA next-generation sequencing

Germline DNA sequencing was performed on DNA extracted from blood samples using ‘Agilent technologies© SureSelect QXT kit’ by targeted Next Generation Sequencing (NGS) on the exonic and flanking intronic regions of *the KRIT1*, *MGC4607*, and *PDCD10* genes with a mean depth of 30 reads. We used ‘SeqPilot© SeqNext V 5.2.0’ for data analysis. We searched for large rearrangements by multiplex ligation-dependent probe amplification, and we used ‘MRC-Holland© Coffalyser’ software for data analysis.

### Somatic DNA lesion next generation sequencing

We collected all operated CM with available tissue samples (frozen or formalin-fixed, paraffin-embedded [FFPE]) from the GHU-Paris Neuropathological Department; diagnosis was confirmed on standard haematoxylin–eosin (HE) staining, allowing for the selection of the most relevant samples. We extracted total DNA from FFPE or frozen tissue samples using ‘Illumina© SureSelect XTHS, UMI’ capture method. Targeted NGS was performed on rat sarcoma viral oncogene homolog (RAS) and phosphatidylinositol-3-kinase (PI3 K) pathway genes (i.e. *AK1, AKT2, AKT3, BRAF, EPHB4, RASA1, GNA11, GNA14, GNAQ, KRIT1, HRAS, KRAS, NRAS, MAP2K1, MAP3K3, MTOR, PIK3CA, PIK3R1, PIK3R2, PTEN, TEK, TSC1,* and *TSC2*) with a mean depth of 1000 reads.^44^ Alignment was performed using the Burrows–Wheeler Aligner (BWA) on the Hg19 human genome. Quality checks were performed following the BROAD Institute guidelines [GATK (HaplotypeCaller, UnifiedGenotyper, Samtools, and Freebayes)]. Variant allele frequency above 1% was considered significant. ProteinPaint^45^ was used to draw lollipop plots.

### Statistical analysis

All statistical analyses were performed using RStudio (version 2023.03.1 + 446). Kaplan–Meier analysis was performed to estimate progression-free survival; the log-rank test was performed to compare survival curves; and Cox analysis was performed to estimate the relative risk (‘Surv, ‘survfit,’ and ‘coxph’ of the ‘survival’ library). Categorical variables are expressed as raw values and proportions; Gaussian numerical variables are presented as means ± standard deviations; and quasi-Gaussian numerical variables are presented as means with ranges. We tested proportions using Fisher’s exact test, and compared distributions between numerical variables using Student’s *t*-test. or analysis of variance. The Mann–Whitney U test was used instead of a *t*-test for groups with fewer than 15 individual measurements.

## Supplementary Material

fcaf408_Supplementary_Data

## Figures and Tables

**Table 1:** Characteristics of patients with cerebral cavernous malformation

	Cohort	S-CM	F-CM	RBT-CM
Number of CM	786	186	557	43
Number of patients	250	168	59	23
Age (years)	9.5 ± 5.1	9.9 ± 4.9	7.4 ± 4.9	12.6 ± 3.6
Sex ratio (F/M)	1.41	1.28	2.28	1.0
Follow-up (years)	5.6 ± 3.7	4.3 ± 3.3	6.1 ± 3.7	3.7 ± 2.5
Initial SP.				
Haemorrhage	53 (6.7%)	35 (18.7%)	17 (3%)	2 (4.6%)
Seizure	101 (12.8%)	71 (38%)	30 (5.3%)	1 (2.3%)
Neurological Deficit	26 (3.3%)	23 (12.3%)	3 (0.5%)	0
Raised ICP	32 (4.0%)	20 (10.6%)	10 (1.8%)	3 (7%)
Incidental	633 (80%)	78 (41%)	525 (94%)	39 (90%)
MRI				
Volume	6.7 [0.01–399.5]	17.9 [0.01–399.5]	3.3 [0.01–252.8]	4.1 [0.01–127.1]
Signal SD.	0 ± 1	0.5 ± 1.4	−0.2 ± 0.73	0.27 ± 1.3
EC. Haemorrhage	36 (4.6%)	21 (11.2%)	14 (2.4%)	2 (4.6%)
DVA	78 (9.8%)	76 (40.6%)	1 (0.1%)	1 (2.3%)
Nb, Vol. (ml) Cortical Brain CM	661 (84.1%), 6.0	141 (75.8%), 15.1	488 (87.6%), 3.4	32 (74.4%), 5.4
Nb, Vol. (ml) Basal Ganglia CM	23 (2.9%), 3.7	8 (4.3%), 9.9	14 (2.5%), 0.5	1 (2.3%), 0.1
Nb, Vol. (ml) Cerebellar CM	45 (5.7%), 20.8	9 (4.8%), 101.3	31 (5.6%), 0.7	5 (11.6%), 0.2
Nb, Vol. (ml) Brainstem CM	57 (7.2%), 6.0	20 (10.7%), 7.8	24 (4.3%), 1.7	4 (9.3%), 0.4
Surgical treatment				
Brain	115 (86%)	76 (81.7%)	34 (97%)	6 (100%)
Basal Ganglia	6 (4.4%)	6 (6.4%)	0	0
Brainstem	6 (4.4%)	5 (5.3%)	1 (3%)	0
Cerebellum	7 (5%)	7 (7.5%)	0	0
Genetics				
KRIT1 mut. (patient)	25/58 (43%)	3/20 (15%)	22/36 (61%)	0/2 (0%)
MGC4706 mut. (patient)	1/58 (2%)	0/20 (0%)	1/36 (3%)	0/2 (0%)
PDCD10 mut. (patient)	14/58 (24%)	1/20 (5%)	12/36 (33%)	1/2 (50%)
PIK3CA var. (CM)	56/92 (61%)	42/66 (64%)	13/21 (62%)	1/5 (20%)

CM, cavernous malformation; RBT, radiation beam therapy; (M/F): male/female; SP, symptoms; ICP, intracranial pressure; SD, standard deviation; EC, extracapsular; Nb, number; Vol, volume; mut, mutation; *KRIT1*, Krev interaction trapped protein 1; *MGC4706*, Malcavernin; *PDCD10*, programmed cell death 10; var, variant; *PIK3CA*, phosphatidylinositol 3-kinase, catalytic, alpha.

**Figure 1:**
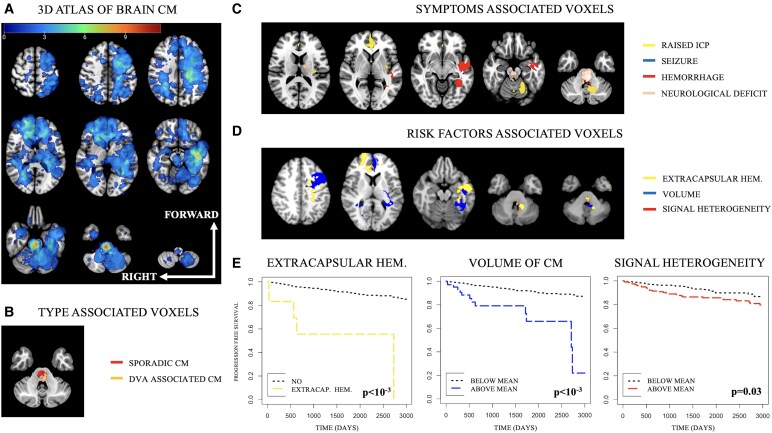
**Radiological assessment of cerebral cavernous malformation: 3D atlas, radiological risk factors, and location-related factors.** (**A**) Frequency of cavernous malformation lesion distribution throughout the brain. Scale bar: number of lesions. (**B**) Voxel-based lesion symptom mapping on type of cavernous malformations and associated developmental venous anomaly. All voxels of each cavernous malformations were included in the analysis. 764 316 (10.6%) of the 7 221 032 voxels were tested: 2336 voxels (red dots) were significantly associated with sporadic cavernous malformation (Liebermeister quasi-exact test, *z_S-CM_* = −1.2 to 5.7 *P* < 0.05) and 1606 voxels (orange dots) with cavernous malformation associated with a developmental venous anomaly (Liebermeister quasi-exact test, *z_DVA_* = −0.9 to 7.3, *P* < 0.05). (**C**) Voxel-based lesion symptom mapping on the initial symptoms. All voxels of each cavernous malformations were included in the analysis. 764 316 (10.6%) of the 7 221 032 voxels were tested: 12 480 voxels (yellow dots) were significantly associated with increased intracranial pressure (Liebermeister quasi-exact test, z_ICP_ = −0.7 to 7.9, *P* < 0.05), 254 voxels (blue dots) with seizure (Liebermeister quasi-exact test, *z_SEIZURE_* = −1.4 to 6.2, *P* < 0.05), 12 622 voxels (red dots) with haemorrhage (Liebermeister quasi-exact test, *z_HEMORRHAGE_* = −0.7 to 7.7, *P* < 0.05), and 10 939 voxels (pink dots) with neurological deficit (Liebermeister quasi-exact test, *z_DEFICIT_* = −0.5 to 8.8, *P* < 0.05). (**D**) Voxel-based lesion symptom mapping on radiological features. All voxels of each cavernous malformations were included in the analysis. 764 316 (10.6%) of the 7 221 032 voxels were tested: 41 370 voxels (yellow dots) were significantly associated with initial extracapsular haemorrhage (Liebermeister quasi-exact test, *z_ECH_* = −0.7 to 8, *P* < 0.05), 28 200 voxels (blue dots) with volume (Liebermeister quasi-exact test, *z_VOLUME_* = −0.9 to 6.7, *P* < 0.05), and 39 voxels (red dots) with signal (Liebermeister quasi-exact test, *z_SIGNAL_* = −1.5 to 3.7, *P* < 0.05). (**E**) Progression-free survival in days for new intracranial haemorrhage of cavernous malformation lesion based on initial extracapsular haemorrhage (*L*, *n* = 32/686 lesions, *P* = 3.10^−8^), size (*L*, *n* = 107/611 lesions, *P* = 2.10^−16^), and signal (*L*, *n* = 263/455 lesions, *P* = 0.03). Groups are divided based on the mean.

**Table 2:** Univariate and multivariate analyses of cerebral cavernous malformation haemorrhagic risk

	Log-rank test	Univariate Cox analysis		Multivariate Cox analysis	
TYPE	**3.10** ^−^ **^11^**				
S-CM		Ref.	—	Ref.	—
F-CM		**1.10** ^−^ **^5^**	−3.5 ± 0.29	0.10	—
RBT-CM		**0.07**	2 ± 0.38	**0**.**04**	2.8 ± 0.5
LOCATION	**0.02**				
Cortical		Ref.	—	Ref.	—
Cerebellar		0.71	—	0.75	—
Basal Ganglia		0.52	—	0.37	—
Brainstem		0.005	2.7 ± 0.34	0.26	—
RADIOLOGY					
EC haemorrhage	**3.10** ^−^ **^8^**	**4.2.10** ^−^ **^6^**	8.6 ± 0.47	0.19	—
Volume	**6.10** ^−^ **^9^**	**1.7.10** ^−^ **^7^**	5.2 ± 0.32	**6.1.10** ^−^ **^5^**	2 ± 0.17
Signal	**0.03**	**0.029**	1.7 ± 0.25	**4.1.10** ^−^ **^3^**	1.4 ± 0.1
DVA	**2.10** ^−^ **^4^**	**3.8.10** ^−^ **^3^**	3 ± 0.31	0.58	—

RBT, radiation beam therapy; Ref, reference for analysis; EC, extracapsular; Signal, T1 signal standard deviation; DVA, development venous anomaly; Bold values indicate statistical significance (*P* < 0.05).

**Figure 2:**
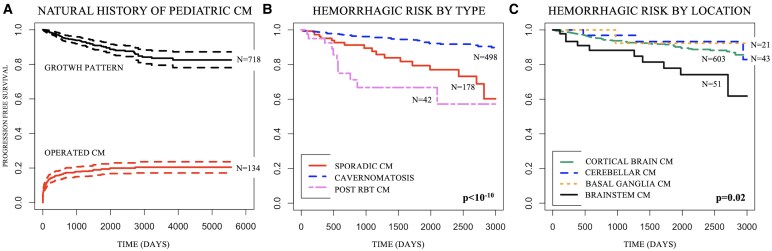
**Natural history of cerebral cavernous malformation.** (**A**) Progression-free survival in days for intracranial haemorrhage after the diagnosis of cavernous malformation in the entire cohort (dots represent haemorrhagic events, *n* = 718 cavernous malformations, *n* = 66 haemorrhagic events, black curve) and cumulative incidence of surgery over time (dots represent surgical excisions, *n* = 718 cavernous malformations, *n* = 134 surgical excisions, red curve). The dotted curves show confidence intervals. (**B**) Progression-free survival in days for intracranial haemorrhage after the diagnosis of cavernous malformation according to the type of cavernous malformations. The red curve indicates S-CM, the blue curve indicates F-CM, and the violet curve indicates RBT-CM (*L*, *n* = 178/498/42 lesions, *P* = 3.10^−11^). (**C**) Progression-free survival in days for intracranial haemorrhage after the diagnosis of cavernous malformation according to the location of cavernous malformations. The blue–green curve represents cortical brain CM, the blue curve represents cerebellar CM, the orange curve represents basal ganglia CM, and the black curve represents brainstem CM (*L*, *n* = 603/43/21/51 lesions, *P* = 0.02).

**Figure 3:**
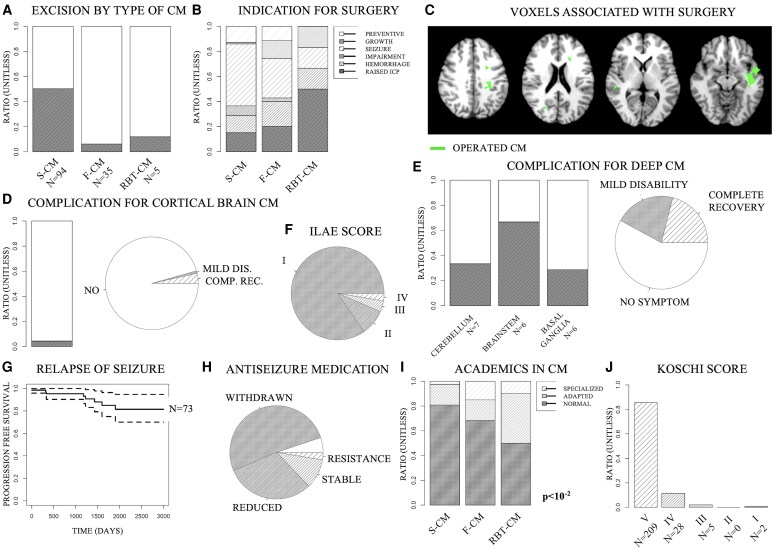
**Surgical planning and results, focusing on epilepsy and global outcomes.** (**A**) percentage of excised cavernous malformation according to the type of lesion (*Fisher’s exact test*, n_S-CM_ = 94/93 lesions, n_F-CM_ = 35/522 lesions, n_RBT-CM_ = 5/37 lesions, *P* = 2.10^−16^). (**B**) Indications for surgery according to the type of cavernous malformation. From top to bottom: preventive surgery, surgery for growth, surgery for seizure, surgery for neurological deficit, surgery for acute haemorrhage, and surgery for raised intracranial pressure (*Fisher’s exact test*, n_S-CM_ = 14/13/7/46/1/12 excised lesions, n_F-CM_ = 7/7/1/11/5/4 excised lesions, n_RBT-CM_ = 3/1/0/1/1/0 excised lesions, *P* = 0.02). (**C**) Voxel-based lesion symptom mapping on excised cavernous malformations. All voxels of each cavernous malformations were included in the analysis. 764 316 (10.6%) of the 7 221 032 voxels were tested: 8399 voxels (green dots) were significantly associated with surgery (Liebermeister quasi-exact test, *z_SURGERY_* = −1.3 to 6.3, *P* < 0.05). (**D**) Complications of cortical brain cavernous malformation surgery (n_COMPLICATIONS_ = 5/115 surgeries). (**E**) Complications of deep cavernous malformation according to location: cerebellum, brainstem, basal ganglia (*Fisher’s exact test*, n_CEREBELLUM_ = 2/5 lesions, n_BRAINSTEM_ = 4/2 lesions, n_BASALGANGLIA_ = 2/4 lesions, *P* = 0.5). (**F**) Postoperative International League Against Epilepsy scores for excised cavernous malformation responsible for focal onset seizures (I = 41/80 patients, Ia = 28/80 patients, II = 7/80 patients, and III−IV = 4/80 patients). (**G**) Kaplan-Meier curve for time to relapse of seizure in days after surgery for cavernous malformation lesion responsible for focal-onset seizures (dots represent patients seizure relapse, *n* = 73 cavernous malformations, *n* = 8 seizure relapse at last follow-up. At 5 years, 85 ± 6% of patients were seizure-free. (**H**) Percentage of patients with antiseizure medication at the final follow-up (n_WITHDRAWN_ = 42/80 patients, n_REDUCED_ = 25/80 patients, n_MAINTAINED_ = 8/80 patients, n_UNKNOW_ = 3/80 patients). (**I**) Percentage of patients requiring school adjustment according to the type of cavernous malformation (S-CM, F-CM, and RBT-CM) (*Fisher’s exact test*, n_S-CM_ = 135/28/4 patients, n_F-CM_ = 40/10/9 patients, n_RBT-CM_ = 10/8/2 patients, *P* = 4.10^−3^). (**J**) King Outcome Scale for Child Head Injury score for patients with cavernous malformation. From left to right: score of V (favourable, *n* = 209/244 patients), IV (*n* = 28/244 patients), III (*n* = 5/244 patients), II (*n* = 0 patient) and I (deceased, *n* = 2/244 patients).

**Figure 4:**
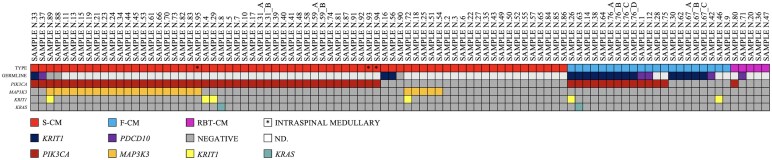
**Germline and somatic molecular diagnosis of operated cerebral and medullar CM.** SAMPLE N*: reference number of CM sample; S-CM, sporadic CM; F-CM, familial CM; RBT-CM, post-radiation beam therapy CM; *KRIT1*, Krev interaction trapped protein 1; *PDCD10*, programmed cell death 10; ND, not determined; *PIK3CA*, phosphatidylinositol 3-kinase, catalytic, alpha; *MAP3K3*, mitogen-activated kinase 3; *KRAS*, Kirsten rat sarcoma viral oncogene homolog.

**Figure 5:**
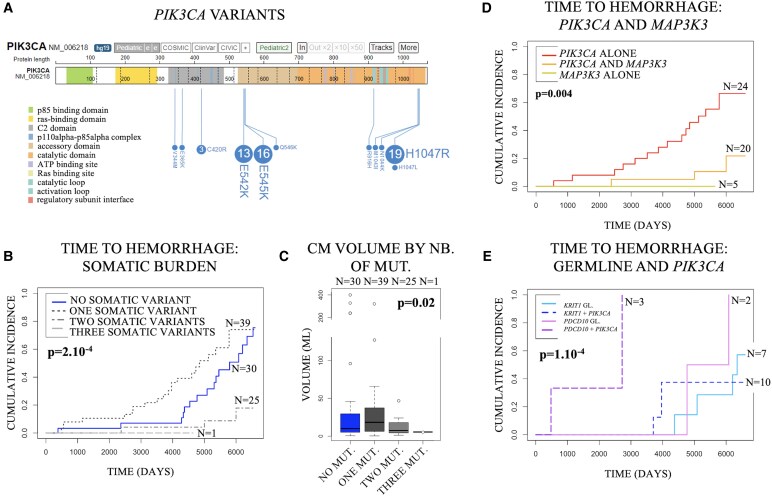
**Molecular diagnosis of operated cavernous malformations and the effect of mutational burden on haemorrhagic risk.** (**A**) The lollipop plots show the locations and number of somatic phosphatidylinositol 3-kinase, catalytic, alpha (*PIK3CA*) variants identified in CM. Obtained from https://pecan.stjude.cloud/. (**B**) Cumulative incidence from birth to first haemorrhage associated with CM depending on the number of somatic variants found in somatic molecular diagnosis (log-rank test, *n* = 30/39/25/1 lesions, *P* = 2.10^−4^). (**C**) Cavernous malformation volume depending on the number of somatic variants found in somatic molecular diagnosis (analysis of variance, *n* = 30/39/25/1 lesions, *P* = 0.02). Each point represents the volume of a cavernous malformation. (**D**) Cumulative incidence from birth to first haemorrhage associated with CM depending on the presence of *PIK3CA* and mitogen-activated kinase 3 (*MAP3K3)* variants found in somatic molecular diagnosis for CM without germline mutation (log-rank test, *n* = 24/20/5 lesions, *P* = 4.10^−2^). (**E**) Cumulative incidence from birth to first haemorrhage associated with CM depending on the presence of *PIK3CA* variants found in somatic molecular diagnosis and the type of Germline mutation found in germline molecular diagnosis (log-rank test, *n* = 7/10/2/3 lesions, *P* = 1.10^−4^).

**Figure 6:**
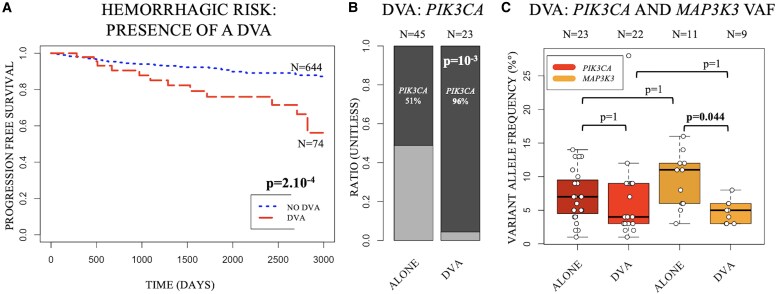
**Natural history of sporadic cavernous malformations, prevalence of *PIK3CA,* and variant allele frequency of *PIK3CA* and *MAP3K3* in sporadic cavernous malformations depending on developmental venous anomaly status.** (**A**) Progression-free survival in days for haemorrhage after diagnosis of cavernous malformation depending on the absence (blue curve) or presence (red curse) of a developmental venous anomaly (*log-rank test*, *n* = 644/74 lesions, *P* = 2.10^−4^). (**B**) Prevalence of a somatic phosphatidylinositol 3-kinase, catalytic, alpha (*PIK3CA)* variant on somatic molecular diagnosis of operated CM depending on the absence (left bar plot) or presence (right bar plot) of a developmental venous anomaly (*Fisher’s exact test*, n_ALONE_ = 22/23 lesions, n_DVA_ = 22/1 lesions, *P* = 1.10^−3^). (**C**) Variant allele frequency of somatic *PIK3CA* and somatic mitogen-activated kinase 3 (*MAP3K3)* variants on somatic molecular diagnosis of operated CM depending on the absence or presence of a developmental venous anomaly (*t values/U*, n_ALONE_ = 23/11 lesions, n_DVA_ = 22/9 lesions, Bonferroni corrected *P* = 1/1/1/0.044). Each point represents the variant allele frequency of *PIK3CA/MAP3K3* variants for each CM lesion.

## Data Availability

Data are available upon reasonable request and institutional approval. The source code used in this paper is available at https://github.com/NeuroSainteAnne/Cavernoma.

## Results

### Cohort description

#### Clinical presentation

Of the 375 screened patients, 257 were included in the analysis, with 250 having intracranial CM and seven having exclusively ISM CM. We distinguished three subgroups: (i) S-CM: *n* = 168 patients with 186 CM, with a mean number of 1.1 CM/patient; (ii) F-CM: *n* = 59 patients with 557 CM, with a mean number of 9.5 CM/patient; (iii) RBT-CM: *n* = 23 patients with 43 CM, with a mean number of 1.9 CM/patient. Table 1 presents the clinical details. In brief, the mean age at diagnosis was 9.5 ± 5.1 years, the male–female ratio was 1.41 and did not vary according to the subgroup (*P* = 0.14). The clinical presentation varied depending on the aetiology of CM: most RBT-CM and F-CM were incidentally diagnosed through imaging (90% and 94% of the cases, respectively). In contrast, S-CM initially presented with seizures (38%), focal deficits (12.3%), or increased intracranial pressure (ICP, 10.6%), whereas 41% were diagnosed incidentally.

#### Radiological presentation

The CM were distributed across the intracranial space as follows: 87% were supratentorial (96.6% cortex, 3.4% basal ganglia) and 23% were infratentorial (55.9% brainstem, 44.1% cerebellum). The mean CM volume in the cohort was 6.7 mL (0.01–399.5) (Table 1 and Fig. S3A). ECH occurred in 4.6%, and 9.8% of CM were satellites of a DVA. This finding was correlated with the CM subgroups because S-CM were larger (17.9 mL) (Fig. S3B), more frequently exhibited ECH (11.2%), and were more often satellites of a DVA (40.6%) than F-CM and RBT-CM (*P* = 8.2.10^−9^, *P* = 1.2.10^−5^ and *P* = 2.2.10^−16^, respectively) (Table 1).

Interestingly, their distribution was not homogeneous because our 3D atlas displayed an overrepresentation of CM in the right frontal pole, left temporal lobe, and brainstem (Fig. 1A). CM location and volume also varied according to aetiology, with brainstem involvement being primarily observed in S-CM (2336 voxels, *z_S-CM_* = −1.2 to 5.7). Brainstem S-CM were also bigger (7.8 versus 1.7/0.4 mL, *P* = 0.01) (Fig. 1B and Table 1).

#### Correlation between clinical symptoms and radiological features

Using VLSM, we further analysed whether the initial symptoms (increased ICP, seizure, haemorrhage, and neurological deficit) were associated with CM location. A higher probability of increased ICP was noted in the frontal interhemispheric areas and within the cerebellum (12 480 voxels, *z_ICP_* = −0.7 to 7.9). The probability of seizure and haemorrhage increased in specific regions: seizures were more likely in the left superior temporal gyrus (254 voxels, *z_SEIZURE_* = −1.4 to 6.2), whereas haemorrhage was more frequently observed in the left temporal lobe, left temporo-occipital junction, and left middle cerebellar peduncle (12 622 voxels, *z_HEMORRHAGE_* = −0.7 to 7.7). Neurological deficits were more frequent in the brainstem (10 939 voxels, *z_DEFICIT_* = −0.5 to 8.8) (Fig. 1C).

#### Correlation between lesion haemorrhagic risk and radiological features

Using VLSM, we further analysed whether radiological features (i.e. DVA, ECH, volume, and signal heterogeneity) were associated with CM location. We first confirmed that the signal standard deviation was similar between T1 imaging with and without injection (−0.375 ± 1 versus −0.412 ± 1; *P* = 0.9).

A higher probability of CM satellites of a DVA was noted in the brainstem (1606 voxels, *z_DVA_* = −0.9 to 7.3). In the case of CM satellites of a DVA, the risk of ICH increased (*HR_DVA_* = 3 ± 0.31, 3.8.10^−3^) (Fig. 1B and Table 2). A higher probability of ECH was noted in the right frontopolar cortex, left premotor area, left temporal lobe, left middle cerebellar peduncle, and brainstem (41 370 voxels, *z_ECH_* = −0.7 to 8). CM with larger volumes were noted in the left interhemispheric cortex, left premotor area, posterior periventricular area, left temporal lobe, and left middle cerebellar peduncle (28 200 voxels, *z_VOLUME_* = −0.9 to 6.7). CM with wider signal heterogeneity were noted only within the brainstem (39 voxels, *z_SIGNAL_* = −1.5 to 3.7) (Fig. 1D). ECH, volume, and signal heterogeneity modulated the risk of ICH. In the case of ECH, the risk of ICH was *HR_ICH_* = 8.6 ± 4 (*P* = 4.2.10^−6^). In case of CM volume above the mean volume of the cohort (6.7 mL), the risk of ICH was *HR_VOLUME_* = 5.2 ± 1.7 (*P* = 1.72.10^−7^). CM signal above the mean standard deviation of the signal intensity of the cohort, the risk of ICH was *HR_SIGNAL_* = 1.7 ± 0.4 (*P* = 0.02) (Fig. 1E and Table 2).

#### Surgery upon diagnosis

Early surgical excision was required for 45 CM in 39 symptomatic children: 20 patients had ICH symptoms, four had neurological deficits, and 15 had focal-onset seizures (mean delay of 3.2 days). Six CM were satellites of a symptomatic CM and were removed during the same surgical procedure. Thirty-four of the 45 CM were S-CM, 10 were F-CM, and one was an RBT-CM. These CM were larger than those in the overall cohort, with a mean volume of 32.7 mL (0.19–399) versus 1.37 mL (0.01–55.7) for the unoperated one (*P* = 8.3.10^−8^) (Fig. S3A).

## Natural history of CM lesions

### CM haemorrhagic risk

Next, we examined the radiological evolution of the CM in patients who underwent multiple MRI scans. Using Kaplan–Meier estimates, we analysed whether haemorrhagic risk could be predicted based on volumetric expansion. Follow-up data were available for 718 CM with a mean follow-up duration of 5.59 ± 3.28 years (Fig. 2A). During diagnosis and at the time of new haemorrhage, CM that underwent excision surgery were larger than those managed conservatively (*P* < 10^−7^ and *P* = 0.01, respectively) (Fig. S3A). We tested the following variables: CM aetiology, CM location, and CM initial radiological characteristics (i.e. ECH, volume, and signal homogeneity assessed using their standard deviation).

The global progression-free survival was 0.976 ± 0.006 at 1 year, 0.904 ± 0.013 at 5 years, and 0.836 ± 0.021 at 10 years; thus, the mean annual lesion risk of haemorrhage was 1.92% (Fig. 2A). CM volumetric expansion was significant, regardless of CM type and location, and the volume did not decrease over time (Fig. S3B). Furthermore, CM volumetric expansion was comparable between cortical and brainstem CM (*P* = 0.18) (Fig. S3C).

Univariate analysis revealed that CM aetiology (F-CM and RBT-CM) (Fig. 2B), CM location (brainstem) (Fig. 2C), CM ECH, CM volume, CM signal (Fig. 1E), and associated DVA were identified (log-rank test) as risk factors for CM haemorrhage (log-rank test: *P* = 3.10^−11^, *P* = 0.02, *P* = 3.10^−8^, *P* = 6.10^−9^, *P* = 0.03, and *P* = 2.10^−4^, respectively; hazard ratios [HRs]: −3.5 ± 0.29, 2 ± 0.38, 2.7 ± 0.34, 8.6 ± 0.47, 5.2 ± 0.32, 1.7 ± 0.25, and 3 ± 0.31, respectively). The multivariate analysis ultimately identified RBT-CM (*P* = 0.04, *HR* = 2.8 ± 0.5), CM volume (*P* = 6.1.10^−5^, *HR* = 2 ± 0.17), and CM signal (*P* = 4.1.10^−3^, *HR* = 1.4 ± 0.1) as predictive of CM haemorrhage (Table 2).

## Cavernous malformation surgery and outcome

### Cavernous malformation surgery

In total, 134 CM were operated on: 94 S-CM, 35 F-CM, and 5 RBT-CM (Fig. 3A). Early surgical excision was performed for 45 CM, whereas 89 CM were followed up before treatment (mean follow-up of 9.8 months after diagnosis) (Fig. 2A).

The indications for surgery (Fig. 3B) were as follows: (i) focal seizures (*n* = 46); (ii) radiological growth (*n* = 23); (iii) preventive surgery (*n* = 11 in the vicinity of eloquent areas); (iv) neurological deficits (*n* = 9). Their locations were mostly supratentorial (*n* = 115 cortical or sub-cortical, *n* = 6 basal ganglia, *n* = 6 brainstem, and *n* = 7 cerebellum), as confirmed by the increased probability of subcortical CM when we tested surgery on VLSM (8399 voxels (*z_SURGERY_* = −1.3 to 6.3) (Fig. 3C). The mean volume of operated CM was 32.7 mL (0.2–399.5) (Fig. S3A).

### Complications

The complication rate was 4.3% (5/115) for supratentorial brain cortical CM (Fig. 3D). The complications included incomplete resection (*n* = 2), neurological focal deficit (*n* = 3), transient hemianopsia (*n* = 1), transient hemiparesis (*n* = 1), and persistent left inferior monoparesis (*n* = 1).

Complications occurred more frequently in deep CM surgery: 33% (2/6) in the basal ganglia, 66% (4/6) in the brainstem, and 28% (2/7) in the cerebellum. These complications included CSF leakage (*n* = 1), subdural collection requiring drainage (*n* = 1), and dyskinesia with partial recovery (*n* = 1). Furthermore, there were four cases of hemiparesis with partial recovery in three cases. One case of worsening tetraparesis was accompanied by acute hydrocephalus, which required shunting (Fig. 3E).

### Epilepsy-related outcome

In total, 80 CM were associated with focal seizures. The mean delay between diagnosis and surgical excision was 245 days (0–2792), with a mean postoperative follow-up of 3.96 ± 3.2 years. The postoperative International League Against Epilepsy scores were I in 51% of cases (41/80), Ia in 35% of cases (28/80), II in 9% of cases (7/80), and III or IV in 5% of cases (4/80, Fig. 3F). Seizure-free survival was 0.85 ± 0.06 at 5 years (Fig. 3G). Two patients later required epilepsy surgery for drug resistance. Antiseizure medications could be withdrawn in 53% of the cases (42/80), reduced in 31% of the cases (25/80), maintained in 10% of the cases (8/80), and unknown in three cases (Fig. 3H).

### Global outcome

In total, 75% of the patients followed a normal academic course, 19% required school adjustment, and 6% required specialized environments. Academic achievements were excellent for patients with S-CM, whereas RBT-CM and F-CM were more prone to require specialized care (40% and 15% of the patients, respectively; *P* = 4.8.10^−3^) (Fig. 3I).

Two patients died: one died from a relapse of a medulloblastoma and the other died from long-term complications following brainstem CM rebleeding. The general recovery outcome evaluated using the KOSCHI score was good (KOSCHI 5, 86%); however, 2% of the patients experienced severe disability (KOSCHI score of 3) (Fig. 3J).

### Intraspinal medullary cavernous malformation

We identified 14 patients with 14 ISM cavernous malformations: 7 F-CM, 6 S-CM, and 1 RBT-CM. The mean age at diagnosis was 8.8 ± 4.7 years. Five of the six patients with ISM F-CM carried a germline *KRIT1* mutation, whereas one carried a germline *PDCD10* mutation.

Diagnosis was incidental for five CM or associated with back pain (one case), pyramidal syndrome (three cases), or paraplegia (five cases). The mean volume was 10.4 mL (2.1–16). Three CM were Zabramski class I, six were class II, two were class III, and three had ECH. Five CM were located in the cervical cord, whereas nine were located in the thoracic cord. After a mean follow-up of 1043 days, the progression-free survival was 0.89 ± 0.11 at 1 year, 0.76 ± 0.15 at 5 years, and 0.25 ± 0.21 at 10 years. The annual haemorrhagic risk was 4.76%. Ten patients underwent surgery without any clinical worsening, five patients recovered to almost symptom-free condition, and one patient partially recovered from initial paraplegia.

## Genomic landscape of CM

### Patients’ germline molecular diagnosis

Fifty-seven of 257 patients (19 S-CM, 36 F-CM, and 2 RBT-CM) underwent germline molecular diagnosis. Germline mutations in *KRIT1* (*n* = 24, 39%), *PDCD10* (*n* = 14, 25%), and *MGC4607* (*n* = 1, 2%) were observed. Three patients with S-CM who developed new unrelated CM underwent molecular diagnosis, which revealed a *KRIT1* mutation in two cases and a *PDCD10* mutation in one case, possibly corresponding to *de novo* variants. The remaining 16 patients were the most recent consecutive S-CM cases for whom systematic germline molecular diagnosis was negative. All but one tested patient with F-CM (*n* = 35) exhibited a loss-of-function germline mutation in either *KRIT1* (22/36), *MGC4607* (1/36), or *PDCD10* (12/36). One patient with multiple RBT-CM exhibited a germline mutation in *PDCD10*. Another patient with multiple RBT-CM had a negative test result. Furthermore, one patient with a unique S-CM who did not undergo germline molecular diagnosis exhibited a *KRIT1* mutation with a variant allele frequency of 50% in the tissue sample molecular diagnosis, strongly suggesting a germline mutation. At the 9-year follow-up, the patient did not develop new CM (Table 1).

### Cavernous malformation somatic molecular diagnosis

In total, 95 CM from 88 patients (69 S-CM from 67 patients, 21 F-CM from 16 patients, and 5 RBT-CM from 5 patients) underwent somatic molecular diagnosis. The targeted NGS analysis identified at least one somatic variant in 65 of the 95 CM (68%) (50/69 S-CM, 14/21 F-CM, and 1/5 RBT-CM) (Fig. 4 and Table S1). Furthermore, 39 CM harboured one somatic variant, 25 CM harboured two variants, and one CM harboured three variants. Fifty-nine of 95 CM (62%) exhibited a gain-of-function mutation in *PIK3CA*, 25 (26%) exhibited an activating missense mutation in *MAP3K3*, six (6%) exhibited *a* loss-of-function mutation in *KRIT1*, and two (2%) exhibited an activating mutation in *KRA*S. Co-occurring somatic variants were observed in 26 of the 95 CM (27%), including *PIK3CA* + *MAP3K3* (*n* = 19), *PIK3CA* + *KRIT1* (*n* = 3), *PIK3CA* + *KRAS* (*n* = 2), *PIK3CA* + *MAP3K3* + *KRIT1* (*n* = 1), and *MAP3K3* + *KRIT1* (*n* = 1) variants. The mutational variant allelic frequency (VAF) ranged from 1% to 28% (*median*: 4%) for *PIK3CA* mutations, from 3% to 16% (*median*: 6%) for *MAP3K3* mutations, from 2% to 25% (*median*: 8.5%) for *KRIT1* mutations, and from 2% to 11% (*median*: 6.5%) for *KRAS* mutations (Table S1). Four of the six patients in whom the somatic *KRIT1* variant was presumed did not undergo germline diagnosis. The variants were classified as somatic based on allele frequencies.

The somatic variants of *PIK3CA* affected the three main hotspots (Fig. 5A and Table S1). The *PIK3CA* p. H1047R variant was the most frequent variant (19/59, 32%), followed by the p. E545 K (16/59, 27%) and p. E542 K (13/59, 22% of cases) variants. Less frequent *PIK3CA* variants were also found: p. C420R (*n* = 3), p. V344M (*n* = 1), p. E365M (*n* = 1), and p. Q546 K (*n* = 1). p. I441M was the sole *MAP3K3* pathogenic variant identified (*n* = 25) (Fig. S4A). Somatic and germline variants of *KRIT1* were all loss-of-function mutations that almost never affected the same positions, except for one variant, and included stop (*n* = 4) and frameshift (*n* = 2) mutations: p. R423* (*n* = 2), p. W322* (*n* = 1), p. E617* (*n* = 1), p. K333fs (*n* = 1), and p. NQ388fs (*n* = 1) (Fig. S4B). Interestingly, p. R423* was associated with *MAP3K3* p. I441M. Other mutations included previously undescribed *KRAS* p. P34Q (*n* = 1) and *KRAS* p. A146 V (*n* = 1) variants, which differed from the classical *KRAS* variants affecting codon p. G12 (Fig. S4C and Table S1). Surprisingly, one patient (ID76) with F-CM who underwent multiple surgeries exhibited the same *PIK3CA* p. E545 K mutations in three of four tested CM samples from different locations, whereas the remaining sample harboured a *PIK3CA* p. E542 K variant (Table S1). Of note, two sisters with F-CM who shared a common germline variant in *KRIT1* (c.1730 + 5G > A) exhibited different somatic *PIK3CA* variants: p. H1047R (ID63) and p. E542 K (ID64).

We then compared the somatic mutational profiles of S-CM, F-CM, and RBT-CM. The incidence of the *PIK3CA* variant did not significantly differ between patients with F-CM and S-CM (64% versus 62%, *P* = 1). Importantly, F-CM did not exhibit co-occurring somatic *PIK3CA* and *MAP3K3* variants, whereas S-CM exhibited co-occurring *PIK3CA* and *MAP3K3* variants in 30% of the cases. Somatic *KRIT1* variants were detected with no significant difference in the incidence between patients with S-CM and F-CM (6% versus 12%, *P* = 0.3). Surprisingly, two CM exhibited co-occurring *MAP3K3* and somatic *KRIT1* variants, one of which also carried a *PIK3CA* variant. Finally, one in five RBT-CM cases exhibited a somatic variant: *PIK3CA* p. C420R variant (Table S1).

### Correlation between mutational status and natural history of CM

We investigated whether mutational status is correlated with the natural history of CM in terms of haemorrhagic risk. Using Kaplan–Meir estimates, we analysed the time from birth to haemorrhage for operated CM (*n* = 95) according to their mutational status. We found that the mutational burden modified the natural history: the risk of haemorrhage increased in CM with one mutation compared with CM negative for somatic diagnosis. However, CM with two or three mutations had a lower risk of haemorrhage than CM negative for somatic diagnosis (*P* = 2.10^–4^) (Fig. 5B). We then analysed the CM volume and found that CM with one somatic variant was the largest, whereas CM without any somatic variant was larger than those carrying two or three somatic variants (*P* = 0.02) (Fig. 5C).

We then observed that for S-CM (excluding S-CM with germline variant), the mutational status of *PIK3CA* and *MAP3K3* modified the relative risk of haemorrhage (*L*, *n* = 24/20/5, *P* = 0.004) (Fig. 5D). S-CM carrying a *PIK3CA* variant alone had the highest risk compared with S-CM carrying both *PIK3CA* and *MAP3K3* variants (*HR* = 0.21 ± 0.65, *P* = 0.01). In contrast, S-CM carrying a *MAP3K3* variant alone exhibited an almost zero risk of haemorrhage (*P* = ∼0).

We observed that in F-CM (including S-CM with a germline variant), the type of germline mutation and the presence of a somatic *PIK3CA* variant modified the risk of haemorrhage, with *PDCD10* CM carrying a somatic *PIK3CA* being the most aggressive (*L*, *n* = 7/10/2/3, *P* = 1.10^−4^) (Fig. 5E).

## Correlation between mutational status and clinical data

### Correlation between mutational status and radiological features

We investigated whether mutations were associated with radiological features. Using VLSM based on mutation status (*PIK3CA*, *KRIT1*, *MAP3K3*, and *KRAS*), we failed to identify specific cerebral locations associated with mutation status. Next, we examined the association between CM and DVA according to mutation status. As previously shown, CM satellites of a DVA carried a higher risk of ICH (*L*, *n* = 644/74, *P* = 2.10^−4^) (Fig. 6A). In total, 96% of CM satellites of a DVA exhibited a *PIK3CA* variant, whereas a *PIK3CA* variant was noted in 51% of the cases of CM alone (*Fisher’s exact test*, N_ALONE_ = 22/23, N_DVA_ = 22/1, *P* = 1.10^−3^) (Fig. 6B). CM alone and CM satellites of a DVA exhibited the same *PIK3CA* VAF (*U,* 7.2% versus 6.1%, *P* = 1), whereas CM alone exhibited higher *MAP3K3* VAF than CM satellites of a DVA (*U,* 9.5% versus 4.8%, *P* = 0.044). *PIK3CA* and *MAP3K3* VAF were similar between CM alone and CM satellites of a DVA (*U, P* = 1 and *P* = 1, respectively) (Fig. 6C). Four patients carried a DVA with multiple satellite CM. In two cases, two CM were operated on in each patient, both harbouring the same *PIK3CA* p. H1047R variant. VAF were 3% and 9% and 3% and 4%, respectively (Table S1). MRI S-CM signal was associated with mutation status: all (25/25) S*-*CM harbouring a *MAP3K3* variant were ranked as Zabramski class II, whereas CM without a *MAP3K3* variant had various Zabramski classes (ZC): *ZCI* = 4, *ZCII* = 15, *ZCIII* = 1, and *ZCV* = 5 (*P* = 6.10^−4^).

## Discussion

### General discussion

In this translational study of 800 paediatric CM, we describe at a large scale the clinical characteristics, natural history, and surgical outcomes of both cerebral and ISM CM in children. We detailed both patient germline and CM somatic genetic profiling and correlated the mutational burden with the natural history of CM lesions. The main findings were as follows: (i) CM has a nonrandom distribution through the brain and exhibits specific location–symptom, location–type, and location–risk factor correlations; (ii) S-CM is revealed by acute symptoms (ICH and epilepsy) related to intra- or extra-capsular haemorrhages; (iii) the annual risk of CM bleeding is 1.9%, stratified by imaging characteristics (i.e. location, extracapsular haemorrhage, volume, signal heterogeneity, and presence of a DVA); and (iv) the overall surgical outcomes of paediatric CM are good but highly associated with CM location. Surgery for supratentorial cortical CM has excellent seizure outcomes and low morbidity; (v) *PIK3CA* is the main somatic variant in S-CM, whereas S-CM harbouring a *MAP3K3* variant is associated with decreased haemorrhage risk. *KRIT1* mutation is a more frequent germline mutation in F-CM, although *PDCD10* CM exhibited a more aggressive course, whereas *MGC4607* was seldomly found in paediatric patients. *PDCD10* CM harbouring a somatic *PIK3CA* variant in F-CM have an increased relative haemorrhagic risk.

### Limitations

Our study has several limitations that should be acknowledged. First, as a single-centre retrospective study, recruitment and classification biases may have occurred; these issues were limited by using a data warehouse. Second, the radiological diagnosis of CM was restricted to CM visible on gradient echo and T1-weighted imaging, thereby excluding some rare cases of F-CM and RBT-CM. However, although almost every F-CM lesion harbours decreased intensity on gradient echo, this is not the case for post-RBT lesions where hypointensity may reflect microbleeds, telangiectasia, or calcifications. Our inclusion criteria may explain the discrepancies between our cohort and those in other studies focusing on RBT-CM. However, we believe that this approach prevents false-negative results in the survival outcome.^7^ Third, radiological analysis was performed using 3D T1 imaging, with or without gadolinium enhancement, which may have introduced bias in the signal analysis, despite signal normalization. Moreover, Zabramski scoring was performed by a single operator owing to the poor interobserver validity obtained between two coauthors (SB and BG) on a sample of 200 CM. This led us to incorporate signal heterogeneity as a continuous variable in the statistical model. Germinal genetic testing was not performed on every patient with a familial history of CM, preventing a comprehensive analysis of F-CM. Eventually, NGS was performed on FFPE tissues conserved over 2 decades, possibly decreasing the sequencing sensitivity. This was overcome by the exclusion of poor-quality samples and the realization of a second NGS after block punching/microdissection in the case of a negative initial NGS. Our somatic gene panel lacks *MGC4607* and *PDCD10* and several genes newly described in CM pathogenesis, such as *MAP2K7* and *GJA4.*^46,47^

### Interpretation

First, we describe the natural history of CM lesions in a large cohort of 257 children with 800 CM over 2 decades, mixing S-CM, F-CM, and RBT-CM. We showed that patients with S-CM were more frequently symptomatic (60% of the cases) than those with F-CM and RBT-CM, where symptoms occurred in <10% of the cases. Using VLSM, we correlated CM location with symptoms and radiological characteristics (i.e. extracapsular haemorrhage, volume, signal, and DVA) and demonstrated that CM are not randomly located in the brain. There are several possible explanations for this result: (i) the diagnosis is more frequent in cases where CM are located in more eloquent areas; (ii) the brain exhibits different susceptibility depending on the anatomical region; and (iii) the uneven prevalence of CM in the brain is associated with their pathogenesis. Furthermore, radiological follow-up over a period of 67 ± 44 months allowed us to stratify the risk of CM haemorrhage. We reported an annual haemorrhagic risk of 1.9%, which is comparable to that observed in adult cohorts (2.4%) but lower than that previously published in paediatric series (3.3%, 3.4%, 23.4%, and 15.9% respectively).^1,20-23^ We identified the following as risk factors for haemorrhage: volume and signal, which further stratify outcomes in our cohort. Therefore, our data challenge the dogma that all paediatric CM will bleed and introduce nuances in surgical indications.

Second, in this study, the surgical outcomes are mainly dependent on CM location. We confirmed at a larger scale the low complication rate previously published in supratentorial cortical CM:^37^ here, < 1% of the operated children developed a permanent disability. We confirmed the excellent seizure outcomes after simple lesionectomy in children with CM,^37,48,49^ as 85% of the operated children were seizure-free after 5 years.^50^ This outcome significantly worsens in adults,^51^ where secondary epileptogenesis and larger epileptic networks may explain the failure of CM ablation to treat epilepsy in 25% of the cases.^52^ In contrast, deep CM (basal GG and brainstem) is associated with higher rates of perioperative complications and postoperative sequelae, occurring in more than one-third and more than half of the cases, respectively.^53^ Owing to the aggressiveness of the natural history and the better recovery and outcomes in children, several authors advocate for surgery in the paediatric population at referral centres, describing surgical pathways and safe entry zones in the brainstem.^29,54^ However, deep CM remain highly challenging lesions, and cerebral plasticity in these locations could not be expected, even in children.

Third, somatic NGS confirmed the high prevalence of *PI3KCA* mutations in paediatric CM, as previously described in adult cohorts, regardless of their subgroup (S-CM, F-CM, or RBT-CM). *PI3KCA* variants were found in 60% of patients with S-CM and F-CM in our cohort. The pathogenesis of CM has been recognized in animal models of cavernomatosis as a multi-hit process requiring loss-of-function events in CCM genes (vascular suppressor genes that constrain vessel growth), along with gain-of-function events in *PIK3CA,* which acts as a vessel ‘oncogene.’^12^ Although CCM loss-of-function genes are found in >90% of familial CM, this is not the case in sporadic CM where *PIK3CA* or *MAP3K3* mutations alone are sufficient to trigger CM formation.^30,46^ Here, we confirm that somatic mutations in CCM genes are not required to trigger CM in children. In sporadic CM, *PIK3CA* mutations were observed in 65% of the positive cases, *MAP3K3* mutations were observed in 36% of the cases (in which 80% were associated with *PIK3CA* mutation), and *KRIT1* mutations were noted in only 5.8%. Furthermore, we report a new gene associated with CM: *KRAS*. *KRAS* is a Ras isoform, whose constitutively activated variants, such as those affecting the p. G12 codon, are frequently found in brain arteriovenous malformations.^55^ However, the variants in our cohort (p. P34Q and p. A146 V) are believed to be not associated with activation of the ERK-MEK pathway. *KRAS* p.P34Q is described in Noonan syndrome, a condition rarely associated with CM.^56^  *KRAS* p. A146 V is described in cancer but not yet in vascular diseases.^57^ Interestingly, *KRAS* is associated with distinct vascular malformations, suggesting that intra-axial vascular malformations have overlapping phenotypes, a hypothesis supported by overlapping transcriptomic profiles in single-cell analysis.^58^ Using VLSM and radiological follow-up, we investigated the correlations between the clinical phenotype and somatic mutations. We confirmed that most CM satellites of a DVA carried a *PIK3CA* mutation (96%), as previously published,^33^ suggesting that DVA could serve as a radiological landmark for *PIK3CA*. We found that the association between *PIK3CA* and *MAP3K3* variants decreased the risk of ICH by 4.8 ± 1.5 folds. Furthermore, no patients with CM harbouring an isolated *MAP3K3* mutation exhibited ICH. However, the small sample size (*n* = 5) precludes any generalization. We confirmed that a *PIK3CA* somatic variant is a risk factor for haemorrhage in both S-CM and F-CM in the paediatric population, which is consistent with previously published data.^12,30,32^

Fourth, germline mutation profiling of F-CM confirmed previously published data in children, with an overrepresentation of *KRIT1* and *PDCD10* loss-of-function mutation in 61% and 36% of the cases, respectively.^21^ In contrast, *MGC4607* was rare, with only one case in our series on contrary to adult onset F-CM where *MGC4607* accounts for 20% of cases.^3^ We confirmed that *PDCD10* CM had a more aggressive course than *KRIT1* CM.^3,59^ Somatic molecular diagnosis of the operated F-CM identified *PIK3CA* mutations in 60% of the cases. This finding is consistent with animal models of CM, where CCM gene loss-of-function alone is not sufficient for CM genesis and requires pseudotumoural proliferation through gain-of-function mutations in *PI3 K* pathway genes.^12^

Taken together, our findings highlight that the natural history and treatment modalities for paediatric CM are similar to those of adult-onset cases, except for seizure control, which appears to be more favourable in children.^20,51^ Similarly, our findings on the molecular biology of paediatric CM emphasized the predominance of *PIK3CA* in the pathogenesis and aggressiveness of CM, as observed in adult-onset cases.^12,30^ We also confirmed the involvement of *MAP3K3* in S-CM in children.^32,46^

## Conclusion

We describe the natural history of CM in 257 children with 800 CM. We report an annual haemorrhagic risk of 1.9%, similar to that observed in adult individuals, but modified by radiological features (extracapsular haemorrhage, volume, signal, association with DVA, and location) and CM type. We presented the first 3D MRI atlas of CM, emphasizing specific symptoms and risk factors associated with their locations. Surgery yields excellent general and epileptic outcomes in supratentorial superficial CM unlike deeper locations, which are associated with high morbidity. Molecular diagnosis of 95 operated CM highlighted *PIK3CA* as a factor for aggressiveness in both S-CM and F-CM. Instead, the presence of *MAP3K3* decreased the risk. We confirmed that DVA is a radiological landmark of *PIK3CA*. Considering the general prognosis and the very high treatment morbidity associated with deep CM, there is an urgent need for medical trials because targeted *PIK3CA* inhibitors are available and have proven effective in animal models.
