# Supplementary material for: Clinical and molecular landscape of paediatric cerebral and spinal cavernous malformations
Source: Brain Commun. 2025 Oct 30;7(6):fcaf408. doi: 10.1093/braincomms/fcaf408 (PMC12596198; doi:10.1093/braincomms/fcaf408)
Supplement: fcaf408_Supplementary_Data [file fcaf408_supplementary_data.pdf]

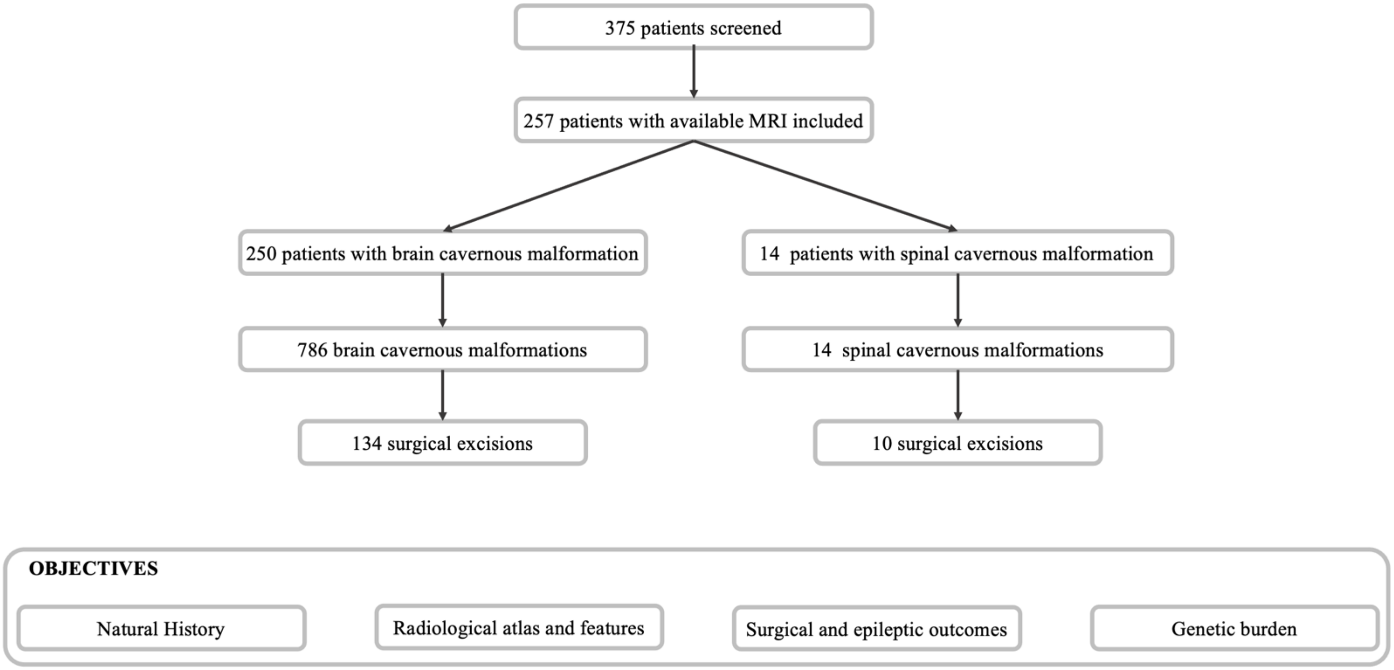

Supplementary Figure 1: Flow chart of patient inclusion and exclusion.

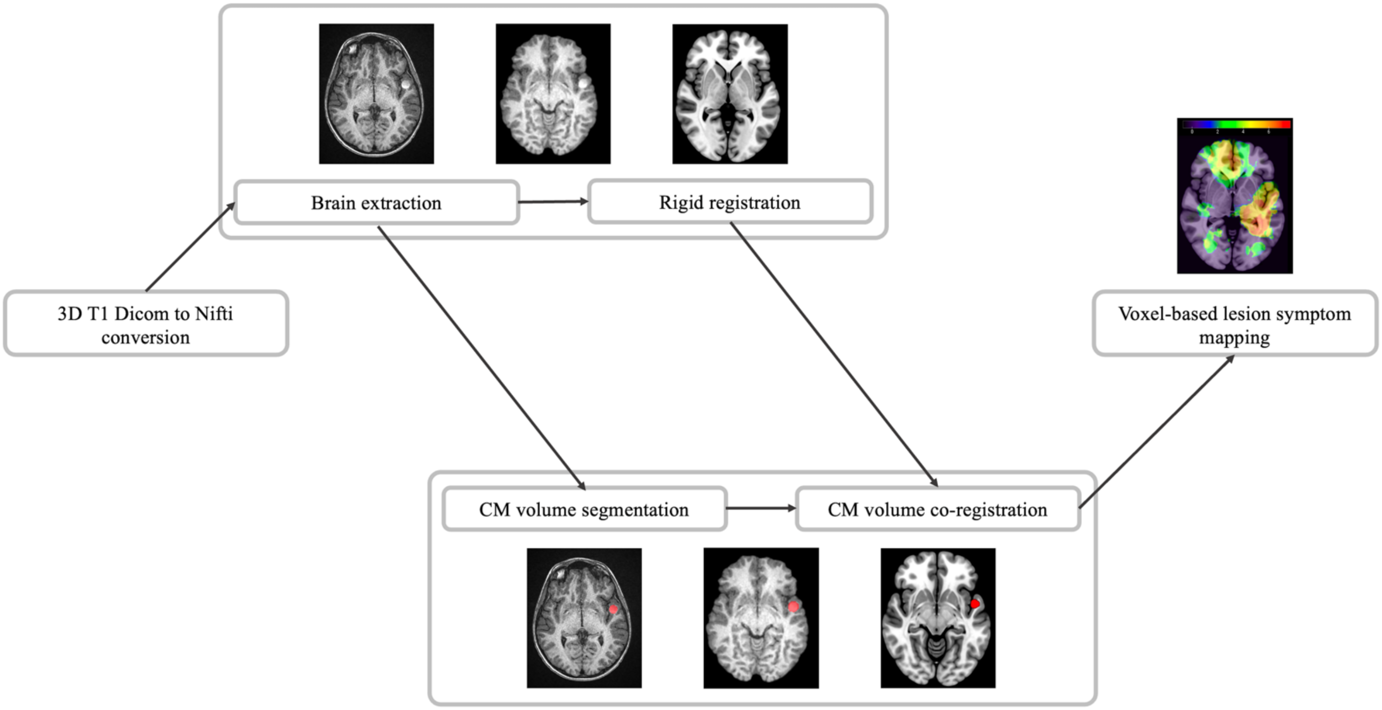

Supplementary Figure 2: MRI normalization, lesion segmentation, and voxel-based lesion symptom mapping scheme. Three-dimensional (3D) digital T1-weighted imaging and communication in medicine (DICOM) format images were converted to the Neuroimaging Informatics Technology Initiative (NIfTI) format. The surrounding brain tissue were suppressed, and the brain volume was rigidly registered on the Montreal Neurological Institute (MNI) space. Simultaneously, CM lesion volumes were segmented and coregistered in the MNI space. Eventually, voxel-based lesion symptom mapping was performed to generate 3D maps. Scale bar: number of lesions.

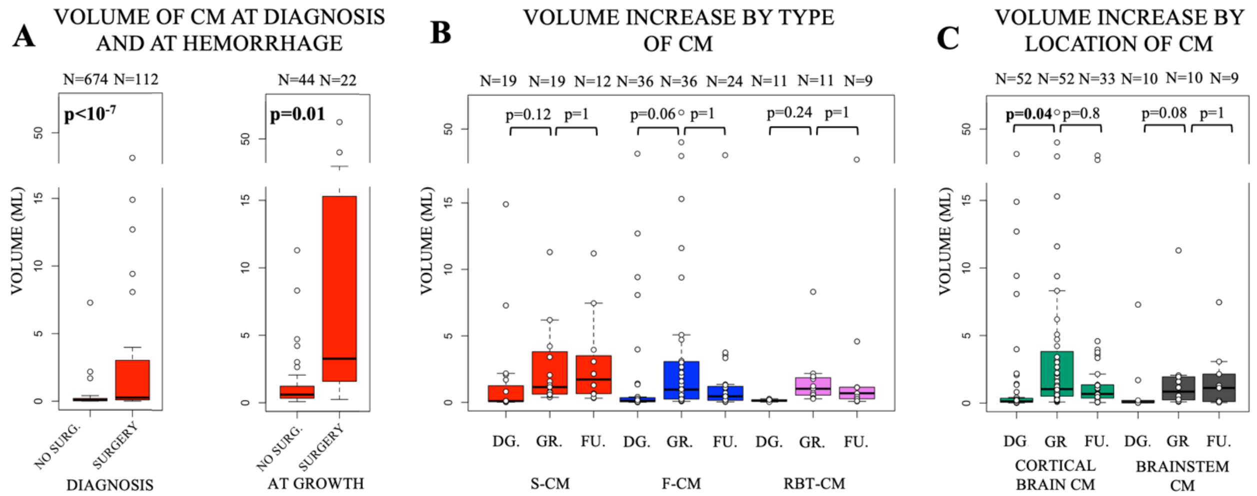

Supplementary Figure 3: Volume increase in operated and unoperated CM, depending on CM type and location. (**A**) Volume in milliliters of brain CM at diagnosis (*t values*, n = 674/112 lesions, p = 8.10^{-8}) and hemorrhage (*t values*, n = 44/22 lesions, p = 0.01) based on surgical status. (**B**) Volume in milliliters of brain CM at diagnosis, growth, and last follow-up according to the type of CM(*t values/U*, $N_{S\text{-}CM}$ = 19/19/12 lesions, Bonferroni corrected p = 0.12/1; $N_{F\text{-}CM}$ = 36/36/24 lesions, Bonferroni corrected p = 0.06/1; $N_{RBT\text{-}CM}$ = 11/11/9 lesions, Bonferroni corrected p = 14/1). Each point represents the volume of each CM lesion. (**C**) Volume in milliliters of brain CM at diagnosis, growth, and last follow-up according to CM location (cortical brain CM or brainstem CM) (*t values/U*, N_{BRAIN} = 52/52/33 lesions, Bonferroni corrected p = 0.004/0.8; $N_{BRAINSTEM}$ = 10/10/9 lesions, Bonferroni corrected p = 0.084/1). Each point represents the volume of each CM lesion. Data for cerebellar and basal ganglia CM are not shown due to an insufficient number of hemorrhagic events (one and three, respectively).

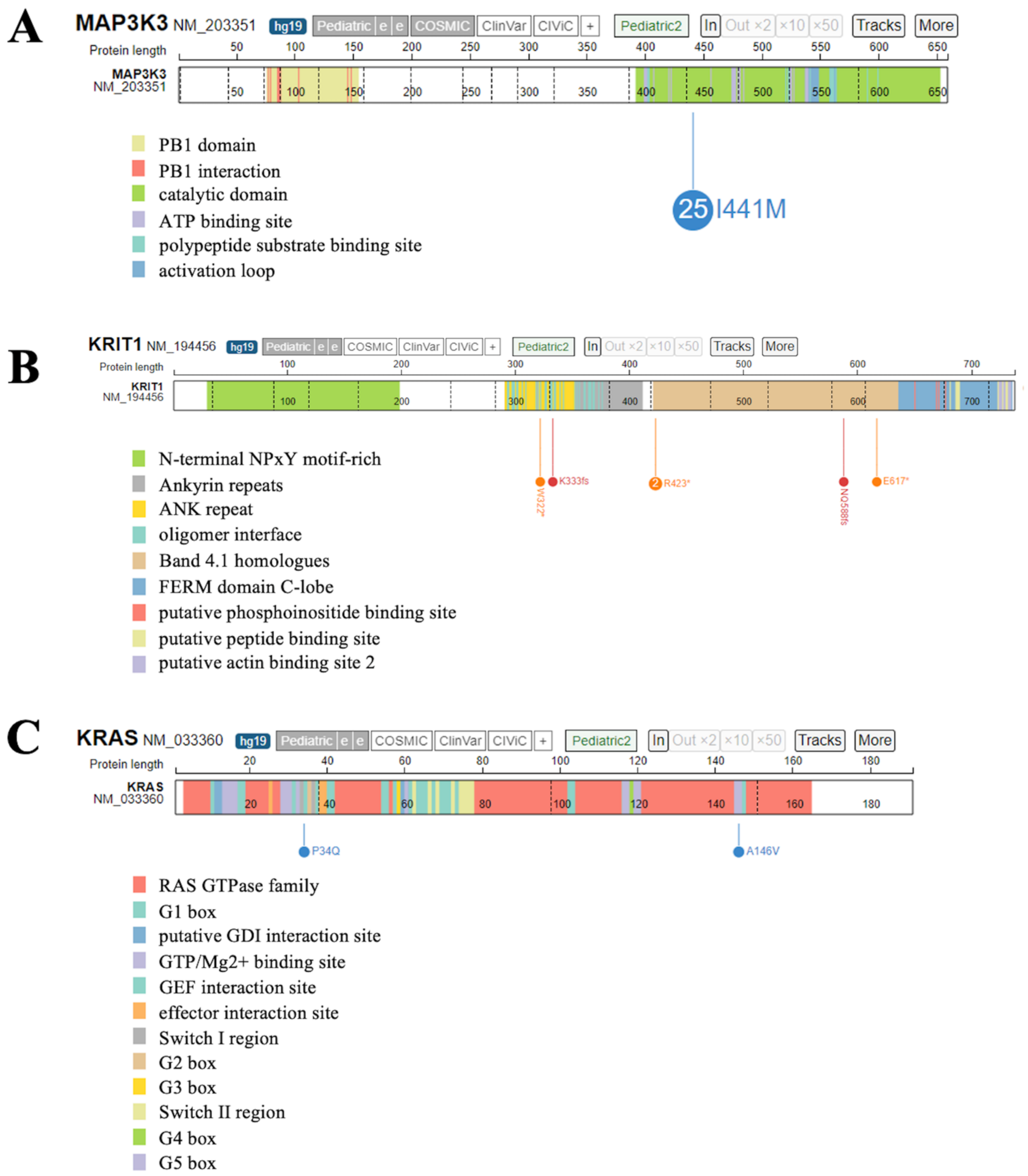

Supplementary Figure 4: Visualization of the somatic variants of *MAP3K3*, *KRIT1*, and *KRAS*. Obtained from https://pecan.stjude.cloud/. The lollipop plots show the locations and number of somatic variants identified in CM; *MAP3K3*, mitogen-activated kinase 3; *KRAS*, Kirsten rat sarcoma viral oncogene homolog; *KRIT1*, Krev interaction trapped protein 1.

Supplementary Table 1: Clinical and molecular features of the operated cerebral and intraspinal medullary CM.

ID	Type	IFS	CM volume (mL)	Germline variant	Som. PIK3CA variant and VAF	Som. MAP3K3 variant and VAF	Som. KRIT1 variant and VAF	Som. KRAS variant and VAF
1	F-CM	Hemorrhage	27.3	*PDCD10* c.150+2T>G	p.E545K 3			
2	S-CM	Seizure	18.9	ND				
3	S-CM	Impairment	45.8	ND				
4	S-CM	Seizure	4.1	ND	p.E365K 3		p.W322* 8	
5	S-CM	Raised ICP	55.8	ND	p.H1047R 3			
6	S-CM	Raised ICP	238.6	ND				
7	S-CM	Hemorrhage	65.9	ND	p.E545K 2			
8	S-CM	Seizure	19.2	ND	p.E542K 14			p.A146V 2
9	F-CM	Raised ICP	28.2	ND				
10	S-CM	Hemorrhage	55.6	ND	p.E545K 3			
11	S-CM	Seizure	23.9	ND	p.E542K 4	p.I441M 4		
12	F-CM	Hemorrhage	22.1	*PDCD10* 3q26.1q26.2	p..H1047R 4			
13	S-CM	Hemorrhage	3.1	ND	p.E545K 3	p.I441M 3		
14	F-CM	Hemorrhage	12.9	*KRIT1* p. K665fs	p.H1047R 7			
15	S-CM	Raised ICP	5.5	ND	p.H1047R 3	p.I441M 6		
16	S-CM	Preventive	0.9	*KRIT1* p. R179*				
17	S-CM	Seizure	21.5	ND	p.H1047R 9			
18	S-CM	Impairment	18.2	ND		p.I441M 10		
19	S-CM	Seizure	4.5	ND	p.H1047R 2	p.I441M 3		
20	RBT-CM	Hemorrhage	9	ND				
21	S-CM	Seizure	2.6	ND	p.H1047R 9	p.I441M 16		
22	S-CM	Seizure	29.5	ND				
23	S-CM	Preventive	7.6	ND	p.E542K 13	p.I441M 12		
24	S-CM	Seizure	9.8	ND	p.E542K 7	p.I441M 6		
25	S-CM	Seizure	44.6	ND		p.I441M 8		
26	F-CM	Seizure	19.4	*KRIT1* p. DQ281fs	p.E545K 5		p.NQ588fs 9	
27	S-CM	Seizure	10.9	ND				
28	F-CM	Seizure	4.9	ND	p.Q546K 1			
29	S-CM	Preventive	1.2	ND	p.E545K 9		p.E617* 15	

30	F-CM	Hemorrhage	29.4	*KRIT1* c.1730+7delAGTA						
31_A	S-CM	Seizure	7.5	ND	p.H1047R	3				
31_B	S-CM	Seizure	1.6	ND	p.H1047R	9				
33	S-CM	Preventive	14.3	*KRIT1* p. N231fs	p.H1047R	4				
34	S-CM	Hemorrhage	1.1	ND	p.E542K	7	p.I441M	5		
35	S-CM	Raised ICP	95.9	ND						
36	RBT-CM	Raised ICP	1.4	ND						
37	S-CM	Preventive	18.8	*PDCD10* c.97-1G>C	p.E542K	9				
38	F-CM	Seizure	37.4	*KRIT1* p. FY686fs	p.H1047R	1				
39	S-CM	Impairment	0.3	ND	p.C420R	1				
40	S-CM	Seizure	8.7	ND	p.E542K	7				
41	S-CM	Raised ICP	320.4	ND	p.H1047R	4				
42	F-CM	Hemorrhage	5.6	*PDCD10* p. E166T						
43	S-CM	Hemorrhage	8.8	ND						
44	S-CM	Seizure	6.6	ND	p.E545K	13	p.I441M	11		
45	S-CM	Seizure	20.4	ND	p.H1047R	4	p.I441M	5		
46	F-CM	Hemorrhage	37.6	ND					p.K333fs	25
47	RBT-CM	Raised ICP	2.5	ND						
48	S-CM	Raised ICP	34.8	ND	p.E545K	11				
49	S-CM	Seizure	5.1	ND						
50	S-CM	Seizure	1.2	ND						
51	S-CM	Seizure	18.2	ND			p.I441M	3		
52	S-CM	Seizure	6.4	ND						
53	S-CM	Seizure	14.9	ND	p.E542K	9	p.I441M	14		
54	S-CM	Preventive	21.9	ND			p.I441M	11		
55	S-CM	Raised ICP	25.4	ND						
56	S-CM	Raised ICP	328.1	*KRIT1* p. Q401fs						
57	S-CM	Preventive	5	ND						
58	S-CM	Seizure	28.8	ND	p.E545K	6				
59_A	S-CM	Seizure	1.4	ND	p.H1047R	3				
59_B	S-CM	Growth	16.9	ND	p.H1047R	4				
61	S-CM	Seizure	3.8	ND	p.E545K	12	p.I441M	6		
62	F-CM	Seizure	7.4	*KRIT1* p. W330*						

63	F-CM	Preventive	6.1	*KRIT1* c.1730+5G>A	p.H1047R	4					p.P34Q	11
64	F-CM	Seizure	48.4	*KRIT1* c.1730+5G>A	p.E542K	4						
65	S-CM	Seizure	23.7	ND								
66	S-CM	Seizure	17	ND	p.H1047R	7	p.I441M	12				
67_A	F-CM	Raised ICP	238	*KRIT1* p. D318fs								
67_B	F-CM	Preventive	26.7	*KRIT1* p. D318fs								
67_C	F-CM	Hemorrhage	0.5	*KRIT1* p. D318fs								
70	S-CM	Hemorrhage	7.4	ND	p.C420R	9	p.I441M	6				
71	RBT-CM	Seizure	1.5	*PDCD10* p. P28Lfs*6								
72	S-CM	Seizure	4.8	ND			p.I441M	14	p.R423*	2		
73	S-CM	Seizure	18.6	ND	p.E542K	10	p.I441M	11				
74	S-CM	Raised ICP	23	ND	p.H1047R	1						
75	F-CM	Hemorrhage	14.3	ND	p.E545K	2						
76_A	F-CM	Hemorrhage	0.2	*KRIT1* p. NL227fs	p.E545K	2						
76_B	F-CM	Raised ICP	0.8	*KRIT1* p. NL227fs	p.E545K	2						
76_C	F-CM	Preventive	2.8	*KRIT1* p. NL227fs	p.E545K	8						
76_D	F-CM	Preventive	6.3	*KRIT1* p. NL227fs	p.E542K	2						
80	RBT-CM	Raised ICP	127.1	ND	p.C420R	2						
81	S-CM	Preventive	14.5	ND	p.E542K	2						
82	S-CM	Impairment	46.7	ND	p.V344M	3	p.I441M	3				
83	S-CM	Preventive	14.2	ND	p.H1047R	7	p.I441M	5				
84	S-CM	Seizure	1.4	ND								
85	S-CM	Raised ICP	5.1	ND								
86	S-CM	Raised ICP	399.6	ND								
87	S-CM	Seizure	1.6	ND	p.E545K	5						
88	S-CM	Seizure	7.8	No variant	p.E542K	9	p.I441M	8				
89	S-CM	Seizure	5.4	No variant	p.E542K	4	p.I441M	3	p.R423*	3		
90	S-CM	Seizure	45.9	No variant								
91	S-CM	Seizure	1.072	ND	p.Q546R	28						
92	S-CM	Hemorrhage	55.856	ND	p.R916H	2						
93	S-CM	Hemorrhage	1.2191	ND	p.H1047R	13						
94	S-CM	Hemorrhage	0.9408	ND	p.M1043IC<br>p.N1044K	5<br>5						
95	S-CM	impairment	1.3266	ND	p.H1047L	5	p.I441M	8				

IFS, indication for surgery; mL, milliliter; som, somatic; VAF, variant allele frequency; *PIK3CA*, phosphatidylinositol 3-kinase, catalytic, alpha; *MAP3K3*, mitogen-activated kinase 3; *KRIT1*, Krev interaction trapped protein 1; *KRAS*, Kirsten rat sarcoma viral oncogene homolog; *PDCD10*, programmed cell death 10; ICP, intracranial pressure; ND, not determined.
